# Effectiveness of zygomatic implants using the externalized technique in the rehabilitation of atrophic maxillae. A systematic review with meta-analysis

**DOI:** 10.4317/medoral.27117

**Published:** 2025-10-14

**Authors:** Helder Lima Rebelo, Pedro Henrique da Hora Sales, Paulo Goberlânio de Barros Silva, Jair Carneiro Leão, Alessandra de Albuquerque Tavares Carvalho

**Affiliations:** 1 DDS, OMFS, MSc,PhD. Oral and Maxillofacial Surgeon, Brazilian Air Force; 2 DDS, OMFS, MSc, PhD. Adjunct Professor, Oral and Maxillofacial Surgery Area, School of Dentistry, Federal University of Alagoas, Brazil; 3 DDS, MSc, PhD. Assistant Professor. Department of Oral Pathology, CHISTHUS university, Fortaleza, CE, Brazil; 4 DDS, MSc, PhD. Full Professor. Department of Clinical and Preventive Dentistry, Federal University of Pernambuco (UFPE), Recife, PE, Brazil; 5 DDS, PhD. Full Professor. Department of Clinical and Preventive Dentistry, Federal University of Pernambuco (UFPE), Recife, PE, Brazil

## Abstract

**Background:**

The objective of this work is to identify the effectiveness and summarize the scientific evidence of zygomatic implants using the externalized technique, as well as to evaluate the possible complications associated with this technique.

**Material and Methods:**

The study was registered in PROSPERO (CRD42022330060) and the searches were carried out in 6 databases (PubMed, Cochrane, LILACS, Scopus, Embase and Google Scholar), by two researchers individually. The aggregated data were subjected to statistical analysis using the MedCalc program for the variables: success rate and frequency of complications, using a 95% confidence interval. The risk of bias of the included studies was determined using the Newcastle-Ottawa Scale (NOS) tool.

**Results:**

912 articles were found in the initial search and 15 of them were included in this systematic review. A total of 1555 zygomatic implants and 1865 conventional implants were part of the study, with an overall success rate of 96.7% for zygomatic implants and 97.9% for conventional implants. There was no statistically significant difference between the survival of zygomatic implants using the externalized technique when compared with conventional implants (*p*=0.015). There was no significant heterogeneity between studies (*p*=0.89, I²=0%). Regarding complications, the most prevalent were sinusitis, which showed a proportion of 3.028% (CI95% = 1.053, 5.980%) and infections, which showed a proportion of 1.56% (CI95% = 0.358, 3.590%). Only three articles included presented a low risk of bias.

**Conclusions:**

Based on the present systematic review and with limited evidence, the use of zygomatic implants using the externalized technique proved to have a high implant success rate and few associated complications for the treatment of atrophic maxillae.

** Key words:**Zygomatic implants, atrophic maxilla, dental implants, zygoma.

## Introduction

In the maxilla, because it has a less dense bone composition and is therefore more prone to major reabsorption, the quality and quantity of bone volume is limited, especially where there is early tooth loss. This condition worsens in the posterior region, interfering with the success rate of dental implants. Depending on the degree of reabsorption of the alveolar ridge, the installation of the dental implant may be compromised [1,2]. Therefore, procedures to increase bone volume are necessary, using bone grafts in order to improve the predictability of oral rehabilitation [3]. The quality and quantity of the remaining bone in the region that is a candidate for receiving dental implants is assessed by many authors using the Cawood and Howell classification [4].

The use of the zygomatic implant (ZI) is an alternative approach to bone reconstruction of the atrophic maxilla. The technique itself consists of anchoring the implant to the body of the zygomatic bone, which is mostly cortical [5-7]. The use of ZIs can shorten the treatment time, reducing the need for prior surgical procedures for bone grafts, and the total number of surgical steps [6,7].

Although the original technique has shown a high success rate for IZs, it also has some disadvantages, such as poor surgical visualization, long surgical time due to the need to lift the sinus membrane, and biological complications such as perforation and sinus pathologies, in addition to functional complications such as poor and/or inadequate emergence profile of the prosthetic abutment due to the palatal positioning of the ZI platform, thus reducing its anchorage in the zygomatic bone [8-12].

In this evolution of the technique for installing ZI, professionals began to be more concerned about the anatomy of the maxillary sinus. In 2006, Migliorança *et al*. proposed installation through an externalized technique, placing it on the lateral wall of the zygomatic maxillary complex to reduce the chances of sinus complications and improve the prosthetic profile [13].

The concept behind the externalized technique is to improve previous techniques by addressing their main disadvantages. The technique is based on longer implants, external to the maxillary sinus and anchored only in the zygomatic bone [13]. It aims to simplify surgical procedures and improve prosthetic results, thus reducing surgical time with good visualization and reducing the risk of sinus pathologies [13].

The objective of this systematic review was to answer the following research question: How effective are zygomatic implants using the externalized technique in the rehabilitation of atrophic maxillae?

## Material and Methods

- Registration and Protocol

The protocol for this study was registered with the International Prospective Register of Systematic Reviews (PROSPERO) under registration number CRD42022330060 and conducted in accordance with the Preferred Reporting Items for Systematic reviews and Meta-Analyses (PRISMA) statement [14].

- Eligibility Criteria

The following inclusion criteria were adopted: 1) randomized clinical trials, non-randomized clinical observational studies that evaluated zygomatic implant studies using the externalized technique; 2) studies conducted in humans with no age or sex restrictions. There were no restrictions regarding language or publication date.

Studies were excluded if they met at least one of the following criteria:

1) study design inadequate for this systematic review (series/case reports, experimental, laboratory, literature reviews, letters to the editor, personal opinions of authors, books/book chapters and conference abstracts);

2) duplicate studies and/or studies that did not report results after the end of the research;

3) studies not peer-reviewed or that have not yet been officially accepted in journals;

4) studies involving patients with poor oral health, with extensive caries, periodontal diseases or infections of pulp or periodontal origin;

- Search Strategy

The search was conducted on the electronic platforms Medline via PubMed

(https://pubmed.ncbi.nlm.nih.gov/), Scopus (https://www.scopus.com/), Cochrane Central Register of Controlled Trials (CENTRAL; https://www.cochranelibrary.com/central/about-central), LILACS (https://lilacs.bvsalud.org/en/), Embase (https://www.embase.com) and Google Scholar (https://scholar.google.com/), by two researchers independently, until July 2024.

For the search on Google Scholar, only the first 300 results were considered [15]. In these databases of scientific articles, a search strategy was inserted with the following descriptors extracted from MeSH: "Zygomatic Implants” [MeSH]), “Atrophic Maxilla” [MeSH]) and “Dental Implants” [MeSH]). An individualized search strategy was adapted for each database following their specific controlled vocabularies, based on appropriate truncations and keyword combinations.

To this end, the search strategy was developed according to the acronym PICOS, whose letters represent:

P- Adult humans with atrophic maxillae;

I - Exteriorized zygomatic implants;

C - Success rate;

O - Fixation rate of zygomatic implants installed in atrophic maxillae. Complications such as: Sinusitis, infections, orbital perforations, oroantral fistula;

S - Randomized or non-randomized clinical trials and observational studies.

- Study Selection

Study selection occurred in two stages. In the first, two independent reviewers (HLR and PHHS) read the titles and abstracts of all the databases searched. This stage was performed using an online application for systematic reviews (Rayyan R, Qatar Computing Research Institute, Doha, Qatar). Articles that did not meet the eligibility criteria adopted were excluded.

In a second stage, the remaining articles were read in full by the same two authors (HLR and PHHS) independently to select those that would comprise this systematic review. A third author (AATC) critically reviewed this stage, and possible disagreements were resolved by consensus. The Kappa test was applied to assess the level of agreement between the authors. Statistical analysis was performed by another researcher (PGBS). A manual search was also performed in the references of the selected articles and in the main scientific journals on oral and maxillofacial surgery.

- Data and Variable Extraction

One reviewer (HLR) extracted data related to the outcomes of interest evaluated in this systematic review and a second reviewer (PHHS) verified all the data collected. The third reviewer (AATC) was consulted to decide on existing disagreements.

Primary Variable: The primary variable was the fixation rate of zygomatic implants installed by the exteriorized technique in atrophic maxillae. This outcome evaluated the success rate of zygomatic implants installed by the exteriorized technique.

Secondary Variable: The secondary variables were complications such as: Sinusitis, infections, orbital perforations, oroantral fistula, peri-implantitis, peri-implant mucositis.

- Assessment of risk of bias

The “Newcastle-Ottawa Scale (NOS)” tool was used to assess the risk of bias. This tool is organized considering three main dimensions of observational studies: participant selection, comparability between groups and exposure criteria for case-control studies, and participant selection, comparability between groups and outcome criteria for cohort studies. After applying these parameters, the studies are classified according to their quality into three different risks of bias: Good, high or very high. This tool assesses the risk of bias through an individual analysis of each variable studied.

- Statistical Analysis

The data were exported and meta-analyzed in the MedCalc software, in which the meta-analysis of odds ratios for the comparison between the success rate of zygomatic and conventional implants, and of combined frequency for assessing the frequency of complications were performed adopting 95%. In both, the inverse variance method by random effects was used and the heterogeneity coefficient I² was calculated. Additionally, the Begg test was used to assess the risk of significant publication bias.

## Results

The search yielded a total of 912 results: 228 in Medline via PubMed, 187 in Scopus, 10 in Central Cochrane, 23 in Lilacs, 166 in Embase and 300 in Google Scholar. Of these, 861 articles were excluded after reading the title, abstract and removal of duplicate articles.

**Figure 1:**
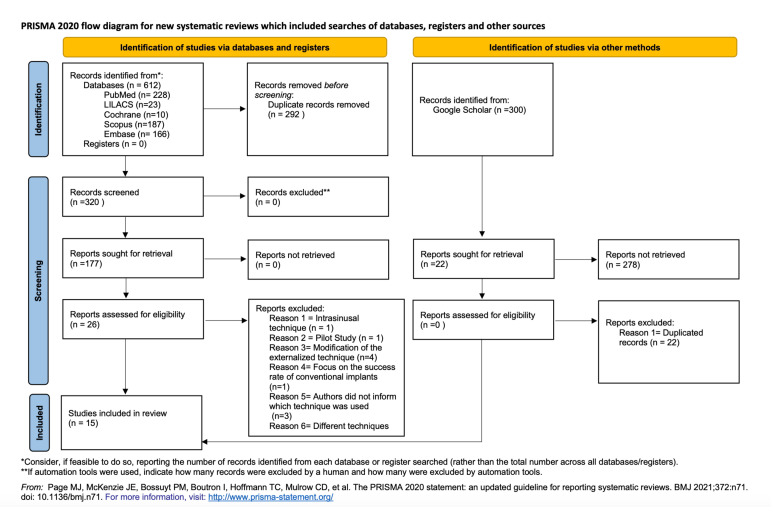
Flowchart of studies included in the review.

The remaining 26 articles were read in full and 11 more were excluded. The reasons for exclusion were: approach using the intrasinusal technique [8] (*n*=1); pilot study [16] (*n*=1); modification of the externalized technique [17-20] (*n*=4); the article focused on the survival rate of conventional implants only [21] (*n*=1); the authors used different techniques to install the zygomatic implants [22] (*n*=1); the authors did not inform which technique was used to install the zygomatic implants [23-25] (*n*=3). At the end of this selection stage, 15 articles were selected for qualitative synthesis and meta-analysis [1,9,10,26-37]. A flowchart containing the details of the selection process is seen in Fig. 1.

- Risk of Bias

The “Newcastle-Ottawa Scale (NOS)” tool was used to assess the risk of bias. After applying the tool criteria, 3 studies presented a low risk of bias, 11 a high risk of bias and 1 a very high risk. (Table 1).

- Demographic Characteristics of the Studies

The included studies totaled a combined sample of 868 patients, with a loss of 71 patients during follow-up. Of these studies, only one (n = 25 patients) did not report the sample according to gender.1 In the end, a total of 586 women and 257 men were reported. Regarding the type of implant used, three studies used only zygomatic implants in the sample [26,29,31]. Regarding follow-up, the studies presented quite heterogeneous values ​​with follow-ups ranging from 6 months to 8 years. These data can be better observed in Table 2.

- Meta-Analysis

The data were exported to the MedCalc software, in which the risk ratio meta-analysis for the comparison between the success rate of zygomatic and conventional implants and the combined frequency meta-analysis for the evaluation of the frequency of complications were performed, adopting 95%. In both, the inverse variance method for random effects was used and the heterogeneity coefficient I² was calculated. Additionally, the Begg test was used to assess the risk of significant publication bias.

- Comparison between the success rate of zygomatic and conventional implants

**Figure 2:**
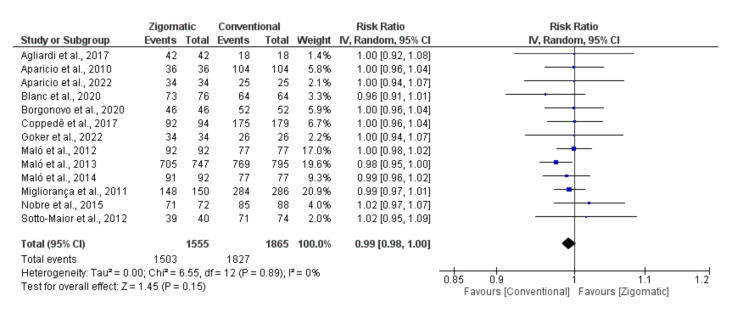
Forest plot comparing the effectiveness of conventional implants versus externalized zygomatic implants.

**Figure 3:**
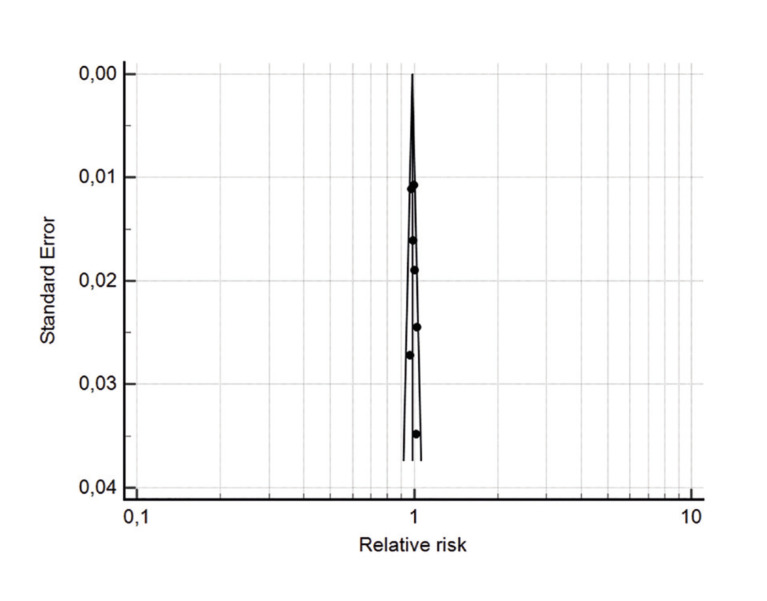
Funnel plot of studies included in the meta-analysis of implant effectiveness.

The comparative meta-analysis between the success rate of zygomatic and conventional implants included a total of 1555 zygomatic implants and 1865 conventional implants, with an overall success rate of 96.7% in zygomatic implants and 97.9% in conventional implants. There was no statistically significant difference between the survival of zygomatic implants using the externalized technique when compared with conventional implants (*p*=0.015) and the success rate of zygomatic implants was 0.99 (CI95% = 0.98, 1.0) times lower than that of conventional implants. There was no significant heterogeneity between the studies (*p*=0.89, I²=0%). The Begg test did not demonstrate a significant risk of publication bias (*p*=0.293) and the distribution of points within the funnel plot was homogeneous (Fig. 2 and Fig. 3).

- Frequency of Complications

**Figure 4:**
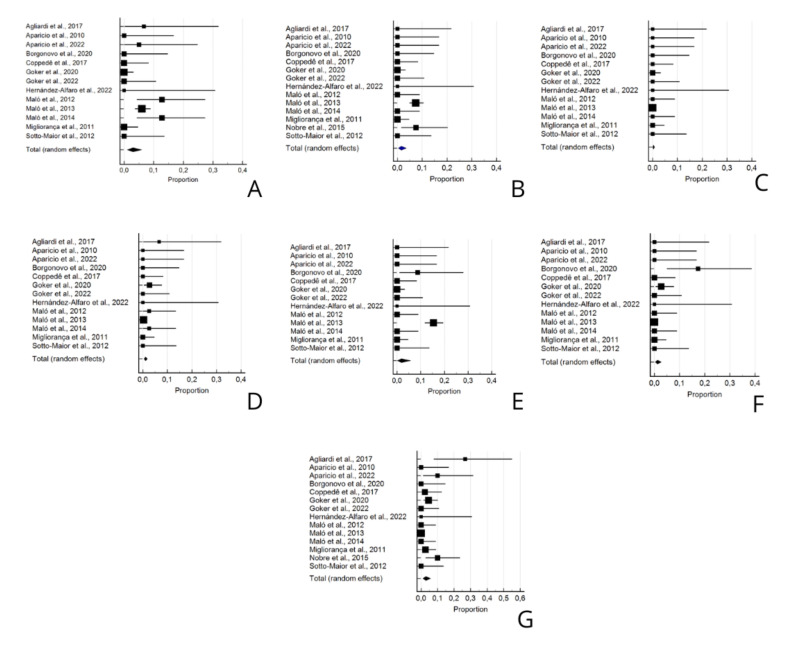
Forest plot of the prevalence analysis of the following variables: A- Sinusitis; B- Infections; C- Orbital Perforations; D- Oro-antral Communication; E- Peri-implantitis; F- Peri-implant mucositis; G- Other complications.

It was possible to perform a meta-analysis of six main types of complications. The frequency of sinusitis was evaluated in 803 patients and showed a proportion of 3.028% (CI95% = 1.053, 5.980%). There was significant heterogeneity between studies (*p*=0.0001, I² = 68.49%) and the Begg test did not demonstrate a significant risk of publication bias (*p*=0.261) (Fig. 4).

The frequency of infections was evaluated in 843 patients and showed a proportion of 1.56% (CI95% = 0.358, 3.590%). There was significant heterogeneity between studies (*p*=0.0005, I² = 64.12%) and the Begg test demonstrated a significant risk of publication bias (*p*=0.012) (Fig. 4).

The frequency of orbital perforations was evaluated in 803 patients and showed a proportion of 0.303% (CI95% = 0.0432, 0.797%). There was no significant heterogeneity between studies (*p*=0.993, I² = 0%) and the Begg test demonstrated a significant risk of publication bias (*p*<0.001) (Fig. 4).

The frequency of oroantral fistulas was evaluated in 803 patients and showed a proportion of 1.073% (CI95% = 0.481, 1.894%). There was no significant heterogeneity between studies (*p*=0.551, I² = 0%) and the Begg test did not demonstrate a significant risk of publication bias (*p*=0.079) (Fig. 4).

The frequency of peri-implantitis was also evaluated in 803 patients and showed a proportion of 1.854% (CI95% = 0.0971, 5.743%). There was significant heterogeneity between studies (*p*<0.001, I² = 86.15%) and the Begg test demonstrated a significant risk of publication bias (*p*=0.0059) (Fig. 4).

Peri-implant mucositis, evaluated in 803 patients, showed a proportion of 1.195% (CI95% = 0.295, 2.691%), with significant heterogeneity between studies (*p*=0.038, I² = 45.31%) and significant risk of publication bias (*p*=0.0027) (Fig. 4).

Other complications were evaluated in 843 patients and showed a proportion of 2.92% (CI95% = 0.996, 5.814%). There was significant heterogeneity between studies (*p*<0.001, I² = 70.64%) and the Begg test did not demonstrate significant risk of publication bias (*p*=0.290) (Fig. 4).

All these results can be better visualized in Table 3 and Fig. 4.

## Discussion

Oral rehabilitation using zygomatic implants in patients with atrophic maxillae is becoming increasingly present as a treatment option.1 In this systematic review, it was found that the success rate of zygomatic implants using the exteriorized technique compared to the installation of conventional implants presented similar results. Exteriorized ZIs proved to be a viable option, with low morbidity, since they do not require prior surgery for bone reconstructions using grafts, and with few complications and a high success rate.

Zygomatic implants and their different techniques are already well documented in the literature, and are increasingly being used as an oral rehabilitation option after the improvement and variations of the original technique initially introduced by Brånemark in 1988 [5].

One of the first studies with a long follow-up period to evaluate the success rate of zygomatic implants installed in atrophic maxillae was published by Brånemark *et al*. in 2004. This study was important to describe the original surgical technique, which consists of two stages: insertion of the implant, through the maxillary sinus, and subsequent anchoring in the zygomatic bone, as well as the installation of conventional implants in the anterior region of the maxilla aiming at better fixation of the dental prosthesis on the implants [5,13].

The externalized technique consists of installing zygomatic implants inserted externally into the maxillary sinus, so that there is no communication with it, remaining in contact with the external surface of its lateral wall, as distally as possible, and preferably in the region of the second premolar or the first upper molar. The IZ is inserted in the lateral portion of the zygomatic-maxillary complex [13,35,36].

The results of this review show that IZs through the externalized technique have clinical results similar to conventional implants when comparing the success rate. A total of 1555 zygomatic implants and 1865 conventional implants, with an overall success rate of 96.7% in zygomatic implants and 97.9% in conventional implants. There was no statistically significant difference between the survival of zygomatic implants using the externalized technique when compared with conventional implants (*p*=0.015).

Kämmerer *et al* 2023 [13], performed a systematic review comparing the success rate of zygomatic implants installed according to the original Brånemark surgical technique, with a total of 923 ZIs, obtaining a survival rate of 90.3-100%, with the zygomatic anatomy guided approach (ZAGA) with a total of 1302 ZIs installed, showing a success rate of 90.4-100% [13], with the success rate of zygomatic implants in both techniques being similar.

It is important to emphasize that for the treatment of atrophic maxillae, the greatest advantage of ZIs with the externalized technique was the avoidance of previous surgeries with bone reconstructions through grafts when compared to the use of conventional implants. As well as the possibility of rehabilitation with the dental prosthesis on the implants immediately, restoring function and aesthetics immediately after the surgical procedure, thus avoiding long treatments with greater morbidity, a fact also noted in the studies by Chrcanovic *et al* 2016. It was observed that late loads were more prone to failure and survival of the ZIs [38].

Even with the high success rate found in this review, the risks inherent to surgical procedures for installing ZI, both systemic and local to patients and prosthetics, may exist, and these possible complications should be well discussed. In the studies by Aparicio *et al* 2014 [37], there was a comparison between the long-term results of the original surgical technique with the Guided Approach to the Anatomy of the Zygoma (ZAGA), and an assessment of the incidence of complications.

The complications analyzed in this systematic review were: sinusitis; infections; orbital perforations; oroantral fistula; peri-implantitis; mucositis; and other complications. The most prevalent were sinusitis, which showed a proportion of 3.028% (CI95% = 1.053, 5.980%) and infections, which showed a proportion of 1.56% (CI95% = 0.358, 3.590%).

It should be noted that the installation of zygomatic implants using the externalized technique has shown a lower incidence of complications when compared with the original surgical technique [13,39,40]. As in the present meta-analysis, sinusitis associated with IZ was the most frequent complication after installation. [38-40].

Among the existing possibilities, sinusitis may result from perforation of the Schneiderian membrane during the procedure, or from lack of fixation of the coronal part of the ZI [31]. Kämmerer *et al* 2023 [13] in their systematic review had a comparison of 9.53% and 4.39% of sinusitis cases in the groups that performed the original surgical technique and ZAGA, respectively. A careful preoperative clinical evaluation and request for imaging exams are necessary for patients with or without a history of sinus pathology before the installation of ZI. In general, infection, mucositis and peri-implantitis are mainly related to inadequate hygiene [1,9,10,24-31]. It is important to note that although this meta-analysis was favorable to the installation of zygomatic implants through the exteriorized technique in patients with atrophic maxillae, some important limitations of this study need to be highlighted.

The main limitations of this study are that most of the included studies were observational. In addition, the risk of bias for most studies was high [11] or very high [1]. Other important limitations that should be mentioned are the sample size of some studies and also the lack of description of gender in one of the studies, which makes statistical analysis difficult and even impossible, as well as a possible meta-analysis of subgroups.

We suggest that future standardized studies evaluating the success rate of zygomatic implants using the externalized technique should follow a methodology that explores greater details related to follow-up, systemic and local conditions of patients, classification of maxillary resorption5 and habits of the target audience, as these factors can have a direct influence on the results. In the present systematic review, the absence of these data was observed in some studies, which may represent a potential risk of bias, especially with regard to complications.

Based on limited evidence, the use of zygomatic implants using the exteriorized technique has proven to be effective and predicTable for the rehabilitation of atrophic maxillae. With a high success rate compared to the installation of conventional implants and a low risk of complications, exteriorized zygomatic implants are an excellent option for treatment. However, it is important to emphasize that new standardized, controlled studies with low risk of bias and long-term follow-up should be conducted in order to increase the scientific evidence.

## Figures and Tables

**Table 1:** Assessment of risk of bias using the NOS tool.

Author and Year	Study Design	Selection	Comparability	Outcome	Total
Sotto-Maior et al, 2012	Prospective cohort	3	1	3	7/9
Maló et al, 2013	Retrospective cohort	2	1	3	6/9
Coppedê et al, 2017	Prospective cohort	2	1	3	6/9
Goker et al, 2020	Retrospective cohort	3	1	3	7/9
Blanc et al, 2020	Retrospective cohort	2	1	2	5/9
Aparicio et al, 2010	Retrospective cohort	2	1	2	5/9
Agliardi et al, 2017	Prospective cohort	2	1	1	4/9
Nobre et al, 2015	Prospective cohort	2	1	2	5/9
Hernández-Alfaro et al, 2022	Prospective cohort	1	1	1	3/9
Maló et al, 2012	Retrospective cohort	2	1	2	5/9
Goker et al, 2022	Retrospective cohort	3	1	3	7/9
Maló et al, 2014	Retrospective cohort	2	1	2	5/9
Aparicio et al, 2022	Prospective cohort	2	1	2	5/9
Borgonovo et al, 2020	Prospective cohort	1	1	2	4/9
Migliorança et al, 2011	Retrospective cohort	1	1	2	4/9

**Table 2:** Summary of results.

Author and Year	Patients	Gender	Age	N of Implants	Nº of Implants loss	Follow -Up (Y)	Marginal boné loss	Bleeding on probing	Success rate
Aparicio et al 2010	20	9 - F 11 - M	44 - 62	36 Exteriorized Zygomatic Implants 104 Conventional implants	0	04	Not reported	Not reported	100% Exteriorized
Migliorança et al 2011	75	48 - F 27 - M	52	150 Exteriorized Zygomatic Implants 286 Conventional implants	02 Zygomatic 02 Conventional	03	Not reported	Not reported	98,7% Exteriorized
Maló et al 2012	39	30 - F 09 - M	53,5	92 Exteriorized Zygomatic Implants 77 Conventional implants	0	03	Reported for Regular Implants only. 1.95±0.66 mm	zygomatic implants showing a bleeding point upon probing.	100%
Sotto-Maior et al 2012	25	8 - M 13 - F	54.14 ± 6.66	40 Exteriorized Zygomatic Implants 74 Conventional implants	1 Zygomatic 3 Conventional	8	Not reported	Not reported	97,5% Exteriorized
Maló et al 2013	352	281 - F 71 - M	55,2	747Exteriorized Zygomatic Implants 795 Conventional Implants	7 Zygomatics 17 Conventional	7	Not reported	15%	94,4% Exteriorized
Maló et al 2014	39	30 - F 09 - M	53,5	92 Exteriorized Zygomatic Implants 77 Conventional Implants	01 Zygomatic	05	For Regular Implants Only 1.16±0.77 mm	Bleeding on probing was recorded in 6 patients (13 implants). Probing pocket depths > 4 mm were present in 13 patients (23 implants) at 5 years of follow-up	98,8% Exteriorized
Nobre et al 2015	40	31 - F 9 - M	56,6	72 Exteriorized Zygomatic Implants 88 Conventional Implants	7 Zygomatic 3 Conventional	2,5	No significant differences No significant difference between the types of Implants.	Not reported	98,6% Exteriorized
Agliardi et al 2017	15	13 - F 2 - M	46-70	42 Exteriorized Zygomatic Implants 18 Conventional Implants	0	7	Reported for Regular Implants - 1.39±0.10 mm	Not reported	100% Exteriorized
Coppedê et al 2017	42	32 - F 10 - M	58	94 Exteriorized Zygomatic Implants 179 Conventional Implants	01 Zygomatic 04 Conventional	3	Exteriorized - 1.34±0.230.23 mm Regular - 1.10± 0.58 mm	Not reported	98,% Exteriorized
Blanc et al 2020	25	Not reported	Not reported	76 Exteriorized Zygomatic Implants 64 Conventional Implants	3 Zygomatics	1,5	Not reported	Not reported	96,1% Exteriorized
Borgonovo et al 2020	23	13 - F 10 - M	63,8 ± 8,9	46 Exteriorized Zygomatic Implants 52 Conventional Implants	0	01	Zygomatic Implants - No Loss Regular Implants - 1.11±0.23 mm	0: 61/98 (62.2%) 1: 37/98 (37.8%) Regular and IZ 0 - No bleeding 1 - Bleeding on gentle probing 2 - Spontaneous bleeding	100% Exteriorized
Goker et al 2020	110	60 - F 50 - M	57,35 ± 10,42	302 Exteriorized Zygomatic Implants	01 Zygomatic	7	Not reported	Not reported	98,34% Exteriorized
Aparicio et al 2022	20	11 - F 09 - M	59.2 ± 8.4	34 Exteriorized Zygomatic Implants 25 Intra-sinus Zygomatic Implants Does not mention quantity of regular implants	0	02	Not reported	Not reported	100% Exteriorized
Hernández-Alfaro et al 2022	10	5 - F 5 - M	60,5 ± 4,2	40 Exteriorized Zygomatic Implants	0	01	90% - grade I (Normal); 7.5% - grade II (Visible implant head); 2.5% - grade III (Up to seven exposed exhalations). At 1-year follow-up, 80%, 17.5%, and 2.5% of the implants showed recession corresponding to grades I, II, and III.	04 implants (10%) in T0 (1st MONTH) and six implants (15%) in T1 (12th MONTH)	100% Exteriorized
Goker et al 2022	32	15 - F 17 - M	60,45 ± 8,74	34 Exteriorized Zygomatic Implants 26 Conventional Implants 5 Pterigoyd implants	0	3,5	Not reported	Not reported	100% Exteriorized

**Table 3:** Meta-analysis of the prevalence of complications in zygomatic implants using the externalized technique.

Study		Sample size	Proportion (%)	95% CI	Weight (%)	Test for heterogeneity and risk of bias publication (Begg's test)						
						Q	DF	Significance level	I^2^ (inconsistency)	95% CI for I^2^	Begg's test	
											Kendall's Tau	Significance level
Sinusitis	Agliardi et al., 2017	15	6.667	0.169 to 31.948	5.21	38.082	12	P = 0.0001	68.49%	43.96 to 82.28	0.2614	P = 0.2135
	Aparicio et al., 2010	20	0	0.000 to 16.843	6.09							
	Aparicio et al., 2022	20	5	0.127 to 24.873	6.09							
	Borgonovo et al., 2020	23	0	0.000 to 14.819	6.52							
	Coppedê et al., 2017	42	0	0.000 to 8.408	8.38							
	Goker et al., 2020	110	0	0.000 to 3.298	10.75							
	Goker et al., 2022	32	0	0.000 to 10.888	7.56							
	Hernández-Alfaro et al., 2022	10	0	0.000 to 30.850	4.09							
	Maló et al., 2012	39	12.821	4.297 to 27.430	8.16							
	Maló et al., 2013	352	5.966	3.731 to 8.975	12.25							
	Maló et al., 2014	39	12.821	4.297 to 27.430	8.16							
	Migliorança et al., 2011	76	0	0.000 to 4.738	9.96							
	Sotto-Maior et al., 2012	25	0	0.000 to 13.719	6.78							
	Total (random effects)	803	3.028	1.053 to 5.980	-							
Infections	Agliardi et al., 2017	15	0	0.000 to 21.802	4.6	36.2361	13	P = 0.0005	64.12%	36.44 to 79.75	0.5056	P = 0.0118
	Aparicio et al., 2010	20	0	0.000 to 16.843	5.44							
	Aparicio et al., 2022	20	0	0.000 to 16.843	5.44							
	Borgonovo et al., 2020	23	0	0.000 to 14.819	5.86							
	Coppedê et al., 2017	42	0	0.000 to 8.408	7.74							
	Goker et al., 2020	110	0	0.000 to 3.298	10.29							
	Goker et al., 2022	32	0	0.000 to 10.888	6.9							
	Hernández-Alfaro et al., 2022	10	0	0.000 to 30.850	3.56							
	Maló et al., 2012	39	0	0.000 to 9.025	7.51							
	Maló et al., 2013	352	7.386	4.881 to 10.636	12							
	Maló et al., 2014	39	0	0.000 to 9.025	7.51							
	Migliorança et al., 2011	76	0	0.000 to 4.738	9.42							
	Nobre et al., 2015	40	7.5	1.574 to 20.386	7.59							
	Sotto-Maior et al., 2012	25	0	0.000 to 13.719	6.12							
	Total (random effects)	843	1.56	0.358 to 3.590	-							
Orbital Perfurations	Agliardi et al., 2017	15	0	0.000 to 21.802	1.96	3.2479	12	P = 0.9935	0.00%	0.00 to 0.00	1	P < 0.0001
	Aparicio et al., 2010	20	0	0.000 to 16.843	2.57							
	Aparicio et al., 2022	20	0	0.000 to 16.843	2.57							
	Borgonovo et al., 2020	23	0	0.000 to 14.819	2.94							
	Coppedê et al., 2017	42	0	0.000 to 8.408	5.27							
	Goker et al., 2020	110	0	0.000 to 3.298	13.6							
	Goker et al., 2022	32	0	0.000 to 10.888	4.04							
	Hernández-Alfaro et al., 2022	10	0	0.000 to 30.850	1.35							
	Maló et al., 2012	39	0	0.000 to 9.025	4.9							
	Maló et al., 2013	352	0	0.000 to 1.043	43.26							
	Maló et al., 2014	39	0	0.000 to 9.025	4.9							
	Migliorança et al., 2011	76	0	0.000 to 4.738	9.44							
	Sotto-Maior et al., 2012	25	0	0.000 to 13.719	3.19							
	Total (random effects)	803	0.303	0.0432 to 0.797	-							
Oro-antral Fistula	Agliardi et al., 2017	15	6.667	0.169 to 31.948	1.96	10.7443	12	P = 0.5510	0.00%	0.00 to 51.63	0.3684	P = 0.0796
	Aparicio et al., 2010	20	0	0.000 to 16.843	2.57							
	Aparicio et al., 2022	20	0	0.000 to 16.843	2.57							
	Borgonovo et al., 2020	23	0	0.000 to 14.819	2.94							
	Coppedê et al., 2017	42	0	0.000 to 8.408	5.27							
	Goker et al., 2020	110	2.727	0.566 to 7.764	13.6							
	Goker et al., 2022	32	0	0.000 to 10.888	4.04							
	Hernández-Alfaro et al., 2022	10	0	0.000 to 30.850	1.35							
	Maló et al., 2012	39	2.564	0.0649 to 13.476	4.9							
	Maló et al., 2013	352	0.284	0.00719 to 1.573	43.26							
	Maló et al., 2014	39	2.564	0.0649 to 13.476	4.9							
	Migliorança et al., 2011	76	0	0.000 to 4.738	9.44							
	Sotto-Maior et al., 2012	25	0	0.000 to 13.719	3.19							
	Total (random effects)	803	1.073	0.481 to 1.894	-							
Peri-implantitis	Agliardi et al., 2017	15	0	0.000 to 21.802	6.4	86.6146	12	P < 0.0001	86.15%	77.99 to 91.28	0.5789	P = 0.0059
	Aparicio et al., 2010	20	0	0.000 to 16.843	6.97							
	Aparicio et al., 2022	20	0	0.000 to 16.843	6.97							
	Borgonovo et al., 2020	23	8.696	1.071 to 28.038	7.23							
	Coppedê et al., 2017	42	0	0.000 to 8.408	8.17							
	Goker et al., 2020	110	0	0.000 to 3.298	9.08							
	Goker et al., 2022	32	0	0.000 to 10.888	7.78							
	Hernández-Alfaro et al., 2022	10	0	0.000 to 30.850	5.53							
	Maló et al., 2012	39	0	0.000 to 9.025	8.07							
	Maló et al., 2013	352	15.341	11.739 to 19.538	9.55							
	Maló et al., 2014	39	0	0.000 to 9.025	8.07							
	Migliorança et al., 2011	76	0	0.000 to 4.738	8.81							
	Sotto-Maior et al., 2012	25	0	0.000 to 13.719	7.38							
	Total (random effects)	803	1.854	0.0971 to 5.743	-							
Peri-implant Mucositis	Agliardi et al., 2017	15	0	0.000 to 21.802	4.06	21.9435	12	P = 0.0382	45.31%	0.00 to 71.36	0.6316	P = 0.0027
	Aparicio et al., 2010	20	0	0.000 to 16.843	5.01							
	Aparicio et al., 2022	20	0	0.000 to 16.843	5.01							
	Borgonovo et al., 2020	23	17.391	4.951 to 38.781	5.53							
	Coppedê et al., 2017	42	0	0.000 to 8.408	8.14							
	Goker et al., 2020	110	2.727	0.566 to 7.764	12.82							
	Goker et al., 2022	32	0	0.000 to 10.888	6.89							
	Hernández-Alfaro et al., 2022	10	0	0.000 to 30.850	2.98							
	Maló et al., 2012	39	0	0.000 to 9.025	7.79							
	Maló et al., 2013	352	0	0.000 to 1.043	17.07							
	Maló et al., 2014	39	0	0.000 to 9.025	7.79							
	Migliorança et al., 2011	76	0	0.000 to 4.738	11.05							
	Sotto-Maior et al., 2012	25	0	0.000 to 13.719	5.86							
	Total (random effects)	803	1.195	0.295 to 2.691	-							
Other Complications	Agliardi et al., 2017	15	26.667	7.787 to 55.100	4.94	44.2807	13	P < 0.0001	70.64%	49.39 to 82.97	0.2123	P = 0.2902
	Aparicio et al., 2010	20	0	0.000 to 16.843	5.72							
	Aparicio et al., 2022	20	10	1.235 to 31.698	5.72							
	Borgonovo et al., 2020	23	0	0.000 to 14.819	6.11							
	Coppedê et al., 2017	42	2.381	0.0603 to 12.566	7.74							
	Goker et al., 2020	110	4.545	1.492 to 10.289	9.73							
	Goker et al., 2022	32	0	0.000 to 10.888	7.02							
	Hernández-Alfaro et al., 2022	10	0	0.000 to 30.850	3.92							
	Maló et al., 2012	39	0	0.000 to 9.025	7.55							
	Maló et al., 2013	352	0	0.000 to 1.043	10.96							
	Maló et al., 2014	39	0	0.000 to 9.025	7.55							
	Migliorança et al., 2011	76	2.632	0.320 to 9.185	9.08							
	Nobre et al., 2015	40	10	2.793 to 23.664	7.61							
	Sotto-Maior et al., 2012	25	0	0.000 to 13.719	6.34							
	Total (random effects)	843	2.92	0.996 to 5.814	-							
